# Comparison of Sonodynamic Treatment Set-Ups for Cancer Cells with Organic Sonosensitizers and Nanosonosensitizers

**DOI:** 10.3390/pharmaceutics15112616

**Published:** 2023-11-11

**Authors:** Aleksandar Radivoievych, Svitlana Prylutska, Oliver Zolk, Uwe Ritter, Marcus Frohme, Anna Grebinyk

**Affiliations:** 1 Division Molecular Biotechnology and Functional Genomics, Technical University of Applied Sciences Wildau, Hochschulring 1, 15745 Wildau, Germany; alra9717@th-wildau.de (A.R.); anna.grebinyk@desy.de (A.G.); 2 Faculty of Health Sciences, Joint Faculty of the Brandenburg University of Technology Cottbus-Senftenberg, The Brandenburg Medical School Theodor Fontane and the University of Potsdam, 14476 Potsdam, Germany; oliver.zolk@mhb-fontane.de; 3 Department of Plants Physiology, Biochemistry and Bioenergetics, National University of Life and Environmental Science of Ukraine, Heroyiv Oborony Str., 15, 03041 Kyiv, Ukraine; psvit_1977@ukr.net; 4 Institute of Clinical Pharmacology, Brandenburg Medical School, Immanuel Klinik Ruedersdorf, 15562 Ruedersdorf, Germany; 5 Institute of Chemistry and Biotechnology, Technical University of Ilmenau, 98693 Ilmenau, Germany; uwe.ritter@tu-ilmenau.de; 6 Deutsches Elektronen-Synchrotron DESY, Platanenallee 6, 15738 Zeuthen, Germany

**Keywords:** ultrasound, C_60_ fullerene, berberine, sonodynamic therapy, apoptosis

## Abstract

Cancer sonodynamic therapy (SDT) is the therapeutic strategy of a high-frequency ultrasound (US) combined with a special sonosensitizer that becomes cytotoxic upon US exposure. The growing number of newly discovered sonosensitizers and custom US in vitro treatment solutions push the SDT field into a need for systemic studies and reproducible in vitro experimental set-ups. In the current research, we aimed to compare two of the most used and suitable SDT in vitro set-ups—“sealed well” and “transducer in well”—in one systematic study. We assessed US pressure, intensity, and temperature distribution in wells under US irradiation. Treatment efficacy was evaluated for both set-ups towards cancer cell lines of different origins, treated with two promising sonosensitizer candidates—carbon nanoparticle C_60_ fullerene (C_60_) and herbal alkaloid berberine. C_60_ was found to exhibit higher sonotoxicity toward cancer cells than berberine. The higher efficacy of sonodynamic treatment with a “transducer in well” set-up than a “sealed well” set-up underlined its promising application for SDT in vitro studies. The “transducer in well” set-up is recommended for in vitro US treatment investigations based on its US-field homogeneity and pronounced cellular effects. Moreover, SDT with C_60_ and berberine could be exploited as a promising combinative approach for cancer treatment.

## 1. Introduction

Despite technological and therapeutic advances in cancer care, the number of cancer patients is growing annually. A total of 17 million new cancer cases were diagnosed in 2016 and reached approximately 20 million in 2022, and cancer deaths increased from9 million in 2016 to over 10 million in 2022 [1,2]. Therefore, cancer remains as the second leading cause of death worldwide [3]. Conventional strategies such as chemotherapy, surgery, and radiotherapy struggle with low target selectivity and harmful side effects [4,5,6,7]. The search for more selective and efficient methods has focused on switchable therapeutics such as sonodynamic therapy (SDT), which originated as a photodynamic therapy (PDT) branch. Unlike PDT, where visible and NIR light is used, SDT uses high frequency ultrasound (US) for a non-thermal, non-invasive, and local tumor ablation based on the toxicity induction of specific chemical sensitizers (sonosensitizers). In that way, dual tumor tissue selectivity can be realized through the targeted accumulation of sonosensitizers in tumor tissue and the US treatment of tumor tissue. SDT can be highly selective, minimizing damage to healthy surrounding tissues and reducing side effects commonly associated with traditional therapies [8]. As a non-invasive treatment, it does not require surgical incisions or the removal of tumor tissue, resulting in reduced pain, shorter recovery time, and lower infection risk [5]. Moreover, SDT-initiated reactive oxygen species (ROS) elicit an immune response to target cancer cells [9,10]. SDT can be repeated as needed without cumulative toxicity concerns [11,12]. In addition, SDT can be used with other treatment modalities, including surgery or chemotherapy, and photodynamic, photothermal, or radiation therapy, to achieve synergistic effectiveness, offering potential multi-pronged cancer care [13]. Conventional US-based methods such as high-intensity focused ultrasound (HIFU) therapy rely on focused energy to heat and destroy cancer cells. Ensuring precise control over the extent and distribution of this heat within the tumor can be challenging and can unintentionally damage healthy tissues surrounding the tumor [6]. In addition, concerns of recurrence exist after HIFU treatment [14]. Nevertheless, the standard limitation of all US treatment methods is the difficulty in penetrating through bone and air, which can limit US treatment effectiveness towards some cancers such as osteosarcoma [15].

The principle of SDT is the sonosensitizing activity induction of particular molecules during sonication. The distribution of US waves in liquid leads to the formation of tiny vapor-filled bubbles, which cavitate and collapse. A rapid bubble implosion heats its interior and produces an intense concentration of energy that results in light emission, called sonoluminescence [16,17,18]. Then, sonoluminescence can induce the excitation of sensitizers. After photon absorption, the sensitizer rises to a short-lived excited state, which can be quenched by molecular oxygen to generate ROS [19,20]. Constant intensification of ROS production leads to oxidative stress and apoptosis of treated cancer cells [21,22]. Oxidative stress also causes DNA damage and induces cell cycle arrest within the cancer cell [23]. In addition, the SDT-induced ROS influence the extracellular matrix, blood vessels, and immune cell activity within the tumor [9,11]. SDT-induced ROS trigger an immune response against cancer cells. The damage caused by ROS can release tumor-associated antigens that alert the immune system to the presence of a tumor [9,10,24]. This can potentially lead to the activation of immune cells that target and destroy cancer cells.

A hematoporphyrin was found to be the first molecule to show cytotoxicity increase towards mouse sarcoma 180 and ascites hepatoma 130 cells under the action of 1.92 MHz US [18,25,26]. The sonosensitizing activity was further discovered for other organic molecules [25,27,28,29], as well as metal-containing [30,31,32] and carbon nanoparticles such as nanotubes [33,34], nanoribbons [35], and fullerenes [36,37,38,39]. Nanoparticles offer several advantages in comparison with organic molecules, including, but not limited to, improved bioavailability, smaller efficient drug doses, less toxicity, and targeting of tumor tissue [31,35,40].

The growing number of newly discovered sonosensitizers pushes the SDT field into a need for systemic studies and reproducible in vitro experimental set-ups. Based on the position of transducers and cell culture vessels (wells with cells), four main types of in vitro US set-ups were defined by Hensel et al. [41]: (1) “well on transducer”—well is placed on transducer and coupled by gel; (2) “well on water surface”—well is placed on the water surface in US bath; (3) “sealed well”—well is submerged into US bath; and (4) “Transducer in well”—transducer is directly submersed in well [41]. Table 1 summarizes the US in vitro set-ups for investigating metal and carbon nanoparticles as sonosensitizers towards cancer cells. If an original article lacked a US set-up description, it was excluded from Table 1.

The “well on transducer” is often characterized by the uneven pressure distribution over the cell layer [41]. One of the main disadvantages of the “well on water surface” set-up is the possible high-temperature effect of the US due to considerable liquid–air and plastic–air interfaces that can lead to low reproducibility of results [42]. The “sealed well” is preferable for in vitro SDT experiments because of its minor US reflections and negligible temperature effects. However, to avoid any possible external effects, a well with cells must be free of air bubbles and reliably sealed [41]. The efficiency of the “transducer in well” set-up depends on its variations in the transducer distance and the thickness of a well’s bottom. Despite these variations, it is easy to implement in sterile conditions and achieves high reproducibility [41,42]. One way to overcome the negative impact of liquid–air and plastic–air interfaces for the “transducer in well” could be to position one extra water tank below the well. However, the SDT in vitro studies often lack detailed US set-up descriptions, making generated data difficult to reproduce, compare, and predict.

In the presented research, we aimed to compare two of the most commonly used SDT in vitro set-ups—“sealed well” and “transducer in well”—in one systematic study. We assessed the distribution of US pressure, intensity, and temperature in wells under US irradiation in “sealed well” and “transducer in well” set-ups. Moreover, we studied the applicability of those set-ups for cellular effect analysis based on evaluating cell viability and caspase 3/7 activity, ROS, and ATP levels in cancer cells of different origins treated with two promising sonosensitizer candidates and 1 MHz US. The current study selected one organic (berberine) and one nanomaterial (C_60_) sensitizer as test compounds. Natural alkaloid berberine is a promising agent for cancer chemotherapy and PDT [43]. US energy via sonoluminescence can be absorbed by berberine and C_60_, promoting the molecule transition from its ground state to an excited state. The excited state can transfer its energy to oxygen generating ROS [44,45], which points to berberine and C_60_ as potential sonosensitizers for SDT. Other than sonosensitizing activity, berberine has proapoptotic effects by inhibiting mitochondrial respiration and modulation of signaling pathways related to cell proliferation [46]. Considerable attention is also devoted to C_60_ as a potential regulator of oxidative balance in biological systems. Low toxicity [47,48] and excellent photosensitizing activity [49,50] of C_60_ make it a promising photo- [36,49] and sonosensitizer [36]. The low photobleaching, high quantum yield, photostability, and improved biocompatibility make C_60_ a prospective molecule for cancer treatment applications [49,51,52].

## 2. Materials and Methods

### 2.1. Chemicals

Dulbecco’s modified Eagle’s medium (DMEM), Eagle’s minimum essential medium (EMEM), phosphate-buffered saline (PBS), fetal bovine serum (FBS), penicillin/streptomycin, l-glutamine, and trypsin were obtained from PAN-Biotech GmbH (Aidenbach, Germany). 3-(4,5-dimethylthiazol-2-yl)-2,5-diphenyl tetrazolium bromide (MTT) and dihydroethidium (DHE) were obtained from Biomol GmbH (Hamburg, Germany). Trypan blue and dimethylsulfoxide (DMSO) were obtained from Carl Roth GmbH + Co. KG (Karlsruhe, Germany). Berberine was obtained from Sigma-Aldrich Co. (St. Louis, MO, USA) and dissolved in sterile distilled water. Caspase-Glo^®^ 3/7 Assay Systems and Mitochondrial ToxGlo™ Assay kits were obtained from Promega GmbH (Walldorf, Germany). The pristine C_60_ aqueous colloid solution was prepared by C_60_ transfer from toluene to water using continuous ultrasound sonication [53].

### 2.2. Ultrasound Exposure

A vacuum pump Savant UVS 400A SpeedVac (Thermo Fisher Scientific Inc., Berlin, Germany) was used for degassing water for the US bath. Cancer cells were seeded and incubated with sonosensitizers for 24 h using cell-based assays, as described below.

#### 2.2.1. “Sealed Well” Set-Up

**Figure 1:**
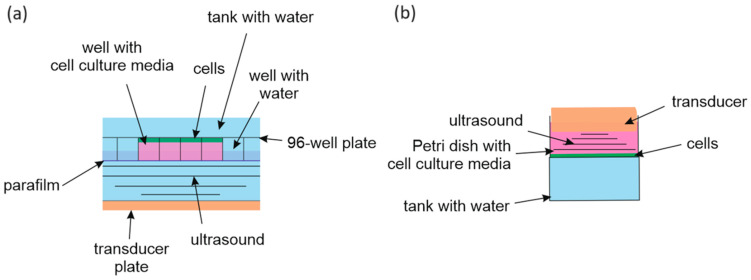
Diagram of the ultrasound exposure equipment: “sealed well” set-up (**a**); “transducer in well” set-up (**b**).

A plate holder was designed and 3D-printed by Oculyze GmbH (Wildau, Germany) for positioning the 96-well plates inside the US bath. The well plate was placed 25 mm above the US transducer. Every empty well was filled with filtered water. Afterward, the plate was covered with parafilm and submerged upside down in the bath (Figure 1a). The US generator 68.101, coupled with an MH2 transducer plate mounted on a water bath (Kaijo, Tokyo, Japan), was used for US treatment. The area and the frequency of the US transducer plate were 92.6 cm^2^ and 950 kHz (~1 MHz), respectively. The US transducer was continuously driven at 100, 150, and 200 W. To compare the two set-ups, US energy (J/cm^2^) was chosen as a unit of US dose. Thus, plates with cells were exposed to 60, 90, and 130 J/cm^2^ of US dose.

#### 2.2.2. “Transducer in Well” Set-Up

Figure 1b schematically shows the “transducer in well” set-up for the US exposure. The US therapy unit DIGI (Strive Enterprises, Yamuna Nagar, India) was driven at 1 W/cm^2^ in continuous wave mode for 30, 60, and 90 s, equivalent to 30, 60, and 90 J/cm^2^ of US dose. To realize the “sealed well” set-up, a tank was filled with filtered water and placed below the Petri dish.

### 2.3. Sonoluminescence Detection

To detect and measure sonoluminescence, an experimental set-up was built. It consisted of the photomultiplier tube (Hamamatsu Photonics, Shizuoka, Japan), connected with the Voltcraft 6150c oscilloscope (Conrad Electronic, Hirschau, Germany) and the Thorn EMI PM28B power supply (Thorn Lighting Ltd., Spennymoor, UK). To enhance the efficiency of sonication and increase sonoluminescence intensity, the US bath was filled with degassed distilled water for better sonication and sonoluminescence intensity [54]. The wells of the plate were filled with experimental liquids: degassed distilled water, distilled water, PBS, and DMEM. Then, the filled plate was placed on the plate holder in the US bath. A polyfoam holder aligned the photomultiplier tube over one of the wells in the 24-well plate.

**Figure 2:**
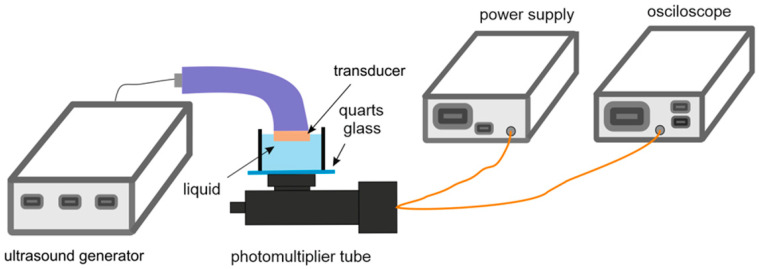
Diagram of the sonoluminescence “transducer in well” set-up.

Sonoluminescence intensity was measured directly in the US “sealed well” set-up described above. To mimic the properties of the “transducer in well” set-up, sonoluminescence measurements were performed in the tank filled with experimental liquid, and the transducer was submersed in the filled tank. This set-up is shown schematically in Figure 2. The set-up was enclosed with aluminum foil to prevent external light from reaching the photomultiplier tube, and measurements were conducted in a dark room.

The data acquired are represented as the average peak-to-peak voltage (Vpp) over a 120 s interval, which quantifies the complete voltage range between the positive and negative peaks in the detected voltage waveform on the photomultiplier tube.

### 2.4. Ultrasound Pressure and Intensity Distribution

For the measurements, a sound field scanner “AIMS III” (Onda Corporation, Sunnyvale, CA, USA), consisting of a water tank equipped with a 3-axis scanner and a “Pico-Scope 5244A” digital storage oscilloscope (DSO) (Pico Technology Limited, St Neots, UK), and an “Imotec” needle hydrophone (Imotec Montagetechnik GmbH, Hausham, Germany) incl. preamplifier (NTR) were used.

The needle hydrophone, including the preamplifier, was calibrated utilizing an “HGL-0200” calibrated capsule hydrophone (Onda Corporation, Sunnyvale, CA, USA) incl. “AH-2010” preamplifier (Onda Corporation, Sunnyvale, CA, USA). The transducers were driven by generators in continuous wave (cw) mode. Burst mode was not possible for both set-ups, and only the phase of the cw-driving signal could be used for triggering the DSO.

The hydrophone was submerged into the tank above the well plate for the “sealed well” set-up and below the well plate for the “transducer in well” set-up. The sensitive tip of the hydrophone was placed on the required positions in the sound field utilizing the 3-axis scanner. The front/back axis of the scanner was assigned to the x-direction, the left/right axis to the y-direction, and the up/down axis to the z-direction.

The following output parameters were measured at particular points in a sound field:

p+: peak compressional acoustic pressure (MPa);
p−: peak rarefactional acoustic pressure (MP);
p_pp_: peak-to-peak acoustic pressure (MPa);

Ita: temporal average intensity (W/m²) (the values of the temporal average intensity Ita are calculated for an assumed cw (continues wave) excitation by dividing the squared amplitude of the pressure wave with 2 and Z (Z = ρc, the acoustic impedance of the propagation medium water)):

$$I_{\mathrm{ta}}=\frac{p_{+}^{2}}{2\mathrm{pc}}$$

### 2.5. Temperature Measurement

The temperature profiles of the plates and Petri dishes were visualized with a Fluke Ti100 infrared (IR) camera (Fluke Corporation, Everett, WA, USA) after sonication in both set-ups with the parameters described above. Obtained pictures were processed and analyzed with Smart View Classic 4.4 (Fluke Corporation, Everett, WA, USA).

### 2.6. Cell Culture

The human cervix adenocarcinoma cell line (HeLa) was generously provided by Dr. Müller (Division of Gastroenterology, Infectiology and Rheumatology, Charité Universitätsmedizin, Berlin, Germany). The Lewis lung carcinoma cell line (LLC) was obtained from (Tebu-Bio GmbH, Offenbach, Germany). The adenocarcinoma human alveolar basal epithelial cell line (A549) and the rat hepatoma cell line (H4llE) were obtained from Hölzel Diagnostika Handels GmbH (Cologne, Germany). HeLa, LLC, and A549 cells were maintained in DMEM. EMEM was used to maintain the H4llE cells. Both cell culture media were supplemented with 10% FBS, 1% penicillin/streptomycin, and 2 mM glutamine. Cells were cultured in 25 cm^2^ flasks at 37 °C with 5% CO_2_ in a humidified incubator binder (Tuttlingen, Germany). A trypsin solution (1:10 in PBS) was used to detach adherent cells. The viable cells were counted upon 0.1% trypan blue staining with a Roche Cedex XS analyzer (Basel, Switzerland).

### 2.7. Cell Viability

Cancer cells were seeded in 96-well plates at 2 × 10^4^ cells per well and in Petri dishes ⌀ = 35 mm at 1.5 × 10^5^ cells per well (both from Sarstedt AG & Co. KG, Nümbrecht, Germany). At 24 h, cells were transferred into 1% FBS DMEM medium containing 20 µM C_60_ or 20 µM berberine. The control cells received treatment with an equal volume of sterile water. After 24 h, treated cells were sonicated with the 1 MHz US with the respective US set-up (Figure 1). To estimate cell viability, cells were incubated for 2 h at 37 °C in the presence of 0.5 mg/mL MTT [55] 24 h after US treatment. The DMSO was used to dissolve the diformazan crystals. The absorbance was measured at 570 nm using a Tecan Infinite M200 Pro microplate reader (Tecan Trading AG, Männedorf, Switzerland). Phase contrast microscopy was carried out with the Keyence BZ-9000 BIOREVO microscope (Keyence Corporation, Osaka, Japan). Images were captured and processed with a BZ-II Viewer (Keyence Corporation, Osaka, Japan).

### 2.8. Intracellular Reactive Oxygen Species Generation

After US exposure, cells were trypsinized, calculated, and transported to a black 96-well plate containing 10^4^ cells per well. To determine ROS production, dihydroethidium (DHE) was applied. A DHE (10 mM) stock solution was prepared in DMSO, stored at −20 °C, and diluted with PBS immediately before use. Cells (10^4^/well) were treated as indicated above and washed once with PBS at 1 h and 3 h of further incubation. DHE (10 µM) was added, and the fluorescence (λex = 495 nm, λem = 585 nm) was recorded with the Tecan Infinite M200 Pro microplate reader.

### 2.9. Intercellular ATP Content

Cells were treated as indicated in the cell viability section. After US exposure, cells were trypsinized, calculated, and transported to a white 96-well plate transferred in 50 µL glucose-free DMEM in a concentration of 10^4^ cells per well. Then, cellular ATP levels were estimated with the mitochondrial ToxGlo™ assay kit (Promega GmbH, Walldorf, Germany) according to the manufacturer’s instructions. The plates were equilibrated to room temperature for 10 min, and 50 µL of the ATP detection reagent was added to each well. The ATP detection reagent consisted of ATP detection containing luciferin, ATPase inhibitors, and thermostable luciferase. After shaking at 600 rpm for 1 min, the Tecan Infinite M200 Pro microplate reader was used to measure the luminescence intensity.

### 2.10. Caspase 3/7 Activity

LLC and HeLa cells were treated as described in the cell viability section. After US exposure, cells were trypsinized, calculated, and transported to a white 96-well plate containing 10^4^ cells per well. The activity of caspases 3/7 was determined during a 4 h period after light exposure using the Caspase-Glo^®^ 3/7 activity assay kit (Promega GmbH, Walldorf, Germany) according to the manufacturer’s instructions. The plates were removed from the incubator and allowed to equilibrate to room temperature for 30 min. After treatment, an equal volume of Caspase-Glo 3/7 reagent was added, followed by gentle mixing with a plate shaker at 300 rpm for 1 min. The plate was then incubated at room temperature for 30 min. The luminescence of each sample was measured with the Tecan Infinite M200 Pro microplate reader.

### 2.11. Statistics

Each experiment was conducted with at least four replicates. Data analysis utilized GraphPad Prism 7 (GraphPad Software Inc., San Diego, CA, USA) and STATISTICA 12 (StatSoft GmbH, Hamburg, Germany). Paired Student’s *t*-tests were used. The threshold for statistical significance was established at *p* < 0.01.

## 3. Results and Discussion

### 3.1. Sonoluminescence Intensity

The optical measurements were conducted using a photomultiplier tube connected to the oscilloscope to assess sonoluminescence intensity. To follow the effect of the chemical composition of the media, the sonoluminescence was measured in degassed distilled water, distilled water, phosphate buffered saline (PBS), and cell culture Dulbecco’s modified Eagle medium (DMEM). The Vpp (peak-to-peak voltage) signal intensity value corresponded to the light reaching the photomultiplier tube. The measurements were conducted in a water bath with 0–5.4 W/cm^2^ output power from the US generator (“sealed well” set-up) and 0–3 W/cm^2^ output power from the US therapy unit (“transducer in well” set-up). The results were normalized using the corresponding Vpp measurements obtained when the shutter of the photomultiplier window was closed and the US was turned on.

**Figure 3:**
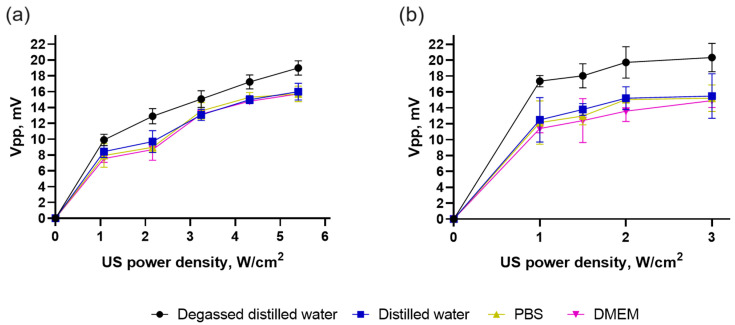
Sonoluminescence intensity in degassed distilled water, distilled water, phosphate buffered saline (PBS), and cell culture Dulbecco’s modified Eagle medium (DMEM): (**a**) in the “sealed well” set-up; (**b**) in the “transducer in well.

The observed rise in the Vpp signal confirmed the presence of sonoluminescence during the propagation of 1 MHz US through liquids in both set-ups (Figure 3). In the “sealed well” set-up, the detected sonoluminescence level increased with the higher output power from the US generator in all tested media. The assessed Vpp evidenced the highest sonoluminescence in the degassed distilled water. The difference in the reduced sonoluminescence level in distilled water, PBS, and DMEM was negligible. A similar tendency was observed in the tank with the submersed transducer at the “transducer in well” set-up. The results agree with our previous study [36] and correlate with other publications [56]. Dissolved gases can become trapped within the bubble during its formation. These trapped gases can affect the dynamics of the bubble’s collapse and significantly alter the conditions required for sonoluminescence to occur [57,58]. Therefore, it was shown that the sonoluminescence intensity in degassed distilled water was considerably higher than in more complex chemical buffers and media (Figure 3). The level of sonoluminescence in the “transducer in well” set-up was substantially higher than in the “sealed well” set-up. In conclusion, the detected sonoluminescence, which occurred during 1 MHz US propagation in the DMEM, can be used for cell-based assays in both set-ups.

### 3.2. Ultrasound Pressure and Intensity Distribution

A hydrophone converts sound vibrations into electrical energy for measurements of sound pressure and temporal average intensity; this directly describe the power and distribution of applied US energy over the well bottom. To check how the US propagated inside the cell culture vessels, sound pressure and temporal average intensity were visualized using the hydrophone in the well plate in a “sealed well” set-up and in a Petri dish in the “transducer in well”. The sound pressure and temporal intensity over the well bottom were estimated to be 1.1, 1.6, and 2.2 W/cm^2^ for the “sealed well” set-up and 1.0 and 3.0 W/cm^2^ of generator output power for the “transducer in well” set-up. Sound pressure and intensity increased in proportion to output power increase.

The average sound pressure and average temporal intensity over the well bottom for both set-ups were estimated and are presented in Table 2.

**Figure 4:**
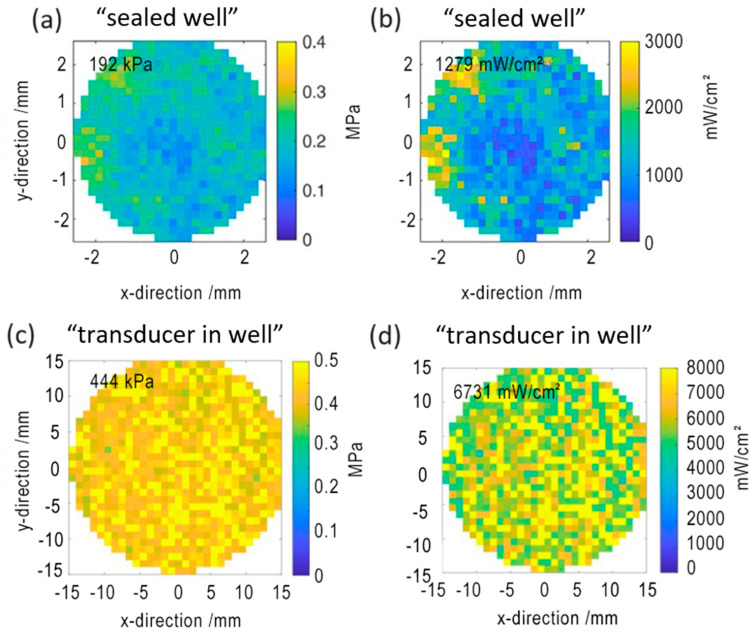
Temporal average intensity (**a**,**c**) and sound pressure (**b**,**d**) amplitude distribution for selected well of 96-well plate, 1 MHz transducer at 150 W, “sealed well”; for chosen Petri dish, 1 MHz transducer at 1 W/cm² “transducer in well” set-up.

The results showed that sound pressure and temporal average intensity were higher on 271 kPa and 5.9 W/cm^2^, respectively, in the “transducer in well” than in the “sealed well” set-up, which could be linked to a shorter distance between the transducer and well bottom—10 mm versus 25 mm, respectively. Obtained images (Figure 4) revealed uneven sound pressure and intensity distribution in a “sealed well” set-up, possibly caused by a complex well-plate structure.

Well plates and Petri dishes, common cell culture vessels for in vitro research, were selected to test “sealed well” and “transducer in well” set-ups based on transducer size. The differences in the detected US pressure and intensity distributions among set-ups pointed to better applicability of the “transducer in well” set-up due to the higher homogeneity of delivered US in the bottom of the cell culture vessel, where cells grow. On the other hand, the set-up designs made hydrophone positioning strictly at the cell-culture vessel bottom with intact bottom plastics impossible, which could introduce some uncertainties. Moderate variation of the growth medium has a negligible effect on the sound pressure and intensity over the well bottom [41,42]. Similar measurements were performed for different set-ups [41,59,60]; however, due to varying methods of irradiation, measurement, and set-up parameters, such data are hard to compare. To increase the reproducibility of sonodynamic in vitro research, more common parameters such as US frequency (Hz), power units (W/cm^2^), propagation mode, duty cycle (%), pressure, and intensity distribution, distance between transducer and well bottom (mm), should be defined.

### 3.3. Temperature Estimation

**Figure 5:**
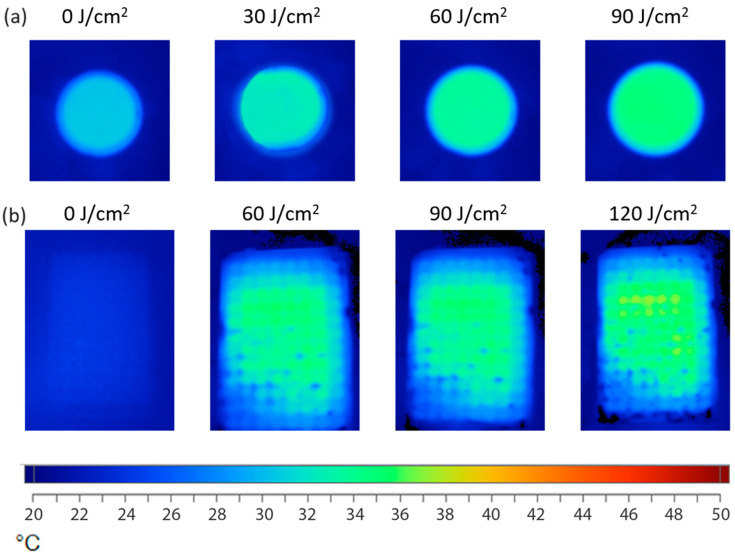
IR-thermography images of the Petri dish after sonication in “transducer in well” set-up (**a**) and the plates after sonication in “sealed well” set-up (**b**).

US energy can be converted into temperature through acoustic heating. The absorbed energy increases the kinetic energy of the particles, leading to an overall temperature rise in the liquid [61,62]. If the temperature exceeds 37 °C, cells undergo destructive changes. Therefore, the IR camera was used to exclude the possible thermal effect of the US. IR thermography images of the plate and Petri dish, filled with distilled water, and exposed to 1 MHz US in “sealed well” and “transducer in well” set-ups are shown in Figure 5a,b, correspondingly. The temperature increase during cell culture vessel exposure to the US was found in both set-ups. The detected maximum temperature in both set-ups did not exceed 36.8 °C after 120 J/cm^2^ sonication, which indicates both set-ups’ applicability for cell-based experiments. The wells in the homogenous temperature distribution were selected for further studies with a “sealed well” set-up.

The temperature distribution in the Petri dish was even, whereas, in the 96-well plate, inhomogeneity was observed. Uneven temperature distribution could result from the well plate’s more complex plastic design. Other studies have shown that temperature insignificantly increased by only 2–3 °C and did not rise above 37 °C during the sonication of cells in different US set-ups [63,64,65]. The critical requirement of cell-based research was met, with demonstrated temperatures kept below 37 °C.

### 3.4. Cell Viability

Cell cultures retain the basic properties of the tumors from which they are derived. Thus, different cell lines have unique parameters of metabolism, gene expression, sensitivity, and treatment resistance, making them integral to in vitro cancer research models. HeLa, LLC, A549, and H4llE were chosen as cell lines of different origins. HeLa and A549 originated from human tissue, while LLC and H4llE from mice and rats. HeLa cells are a standard model because this cell line has been pivotal in numerous scientific discoveries [66]. A549 cells are characterized by strong resistance to oxidative stress [67,68]. Meanwhile, LLC cells are tumorigenic and characterized by rapid proliferation [69]. H4llE cells often metastasize to the lungs, bones, and regional lymph nodes [70].

To follow how physical characteristics of tested in vitro US treatment set-ups manifest in their efficiency for sonodynamic treatment of cancer cells, the toxic effects towards LLC, HeLa, H4llE, and A549 cells were evaluated in both set-ups after incubation with C_60_ or berberine. First, cells were treated with either 20 μM C_60_ or 20 μM berberine for 24 h and then were exposed to 1 MHz US at 0–120 and 0–90 J/cm^2^ for the “sealed well” and “transducer in well” set-ups, respectively. Then, 24 h after US exposure, cell viability was estimated with MTT assay. The viability of the respective control cells without any sonosensitizer and US treatment was considered 100%.

**Figure 6:**
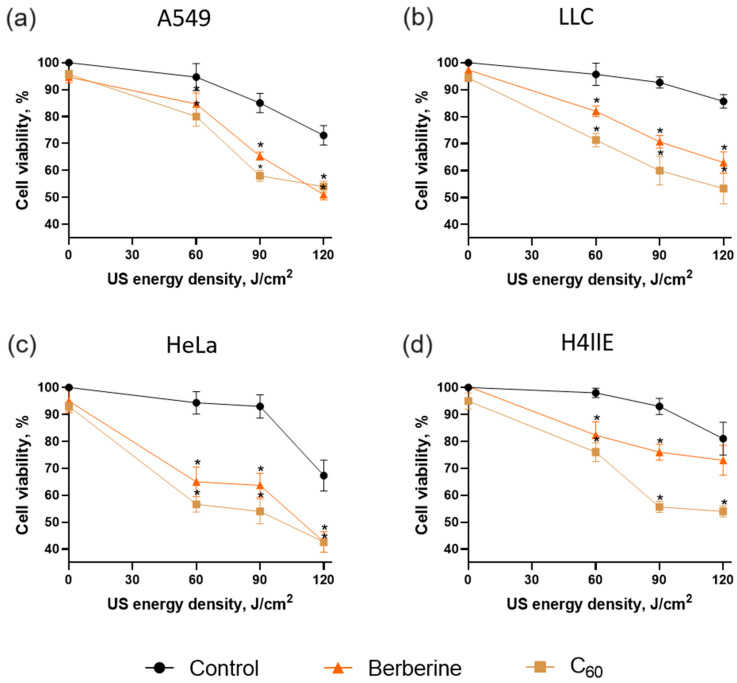
Viability of A549 (**a**), LLC (**b**), HeLa (**c**), and H4llE (**d**) cells incubated in the presence of 20 µM C_60_ or 20 µM berberine and treated with 1 MHz ultrasound (US) in the “sealed well” set-up; *—*p* ≤ 0.01 in comparison with the viability of cells, treated with the respective duration of US.

**Figure 7:**
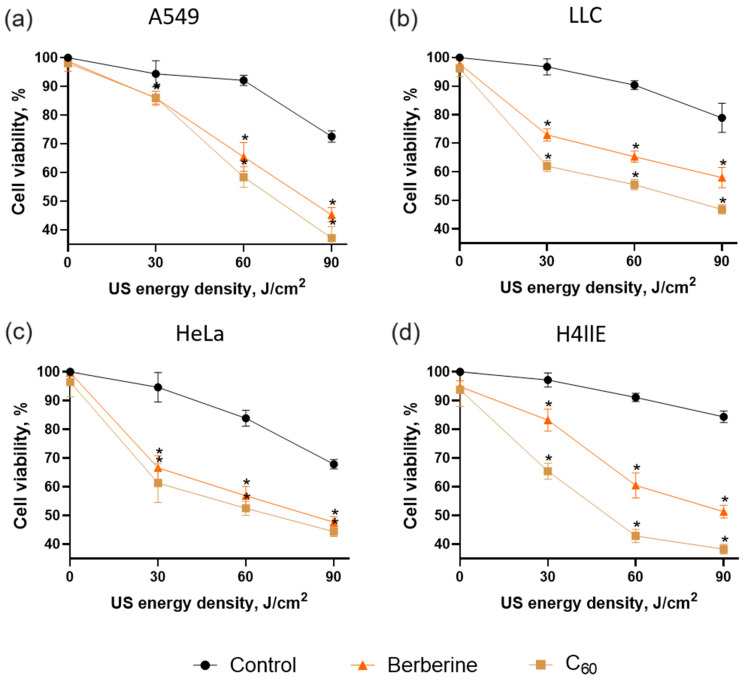
Viability of A549 (**a**), LLC (**b**), HeLa (**c**), and H4llE (**d**) cells incubated in the presence of 20 µM C_60_ or 20 µM berberine and treated with 1 MHz ultrasound (US) in the “transducer in well” set-up; *—*p* ≤ 0.01 in comparison with the viability of cells, treated with the respective duration of US.

The viability analysis is presented for cancer cells treated in the US presence of sonosensitizers using “sealed well” and “transducer in well” set-ups in Figure 6 and Figure 7, respectively. Control cells were incubated with equal sterile water and had no significant viability changes after sonication with low and middle doses. However, the highest US doses above 90 J/cm^2^, which were applied in the experiments, decreased cell viability in both set-ups to ≥67 ± 6%. US cytotoxicity depends on the specific parameters of the US exposure, including intensity, frequency, duration, and mode of operation. US-induced streaming of surrounding liquid and cavitation can directly damage cell membranes, leading to cytotoxic effects [71,72].

All cell lines treated with tested sonosensitizers and US demonstrated a significant decrease in cell viability (Figure 6 and Figure 7). The highest ratio between the control and cells incubated with sonosensitizer was observed after sonication with a middle dose of US (90 and 60 J/cm^2^ in “sealed well” and “transducer in well” set-ups, respectively). Thus, the US dose of 90 J/cm^2^ in the presence of 20 µM C_60_ decreased the cell viability to 58 ± 2%, 60 ± 6%, 54 ± 4%, and 56 ± 2% for A549, LLC, HeLa, and H4llE cells, respectively, in the “sealed well” set-up. Meanwhile, berberine decreased the cell viability of A549, LLC, HeLa, and H4llE cells to 65 ± 2%, 70 ± 2%, 64 ± 4%, and 76 ± 3%, respectively, exposed to US in the same set-up (Figure 6).

**Figure 8:**
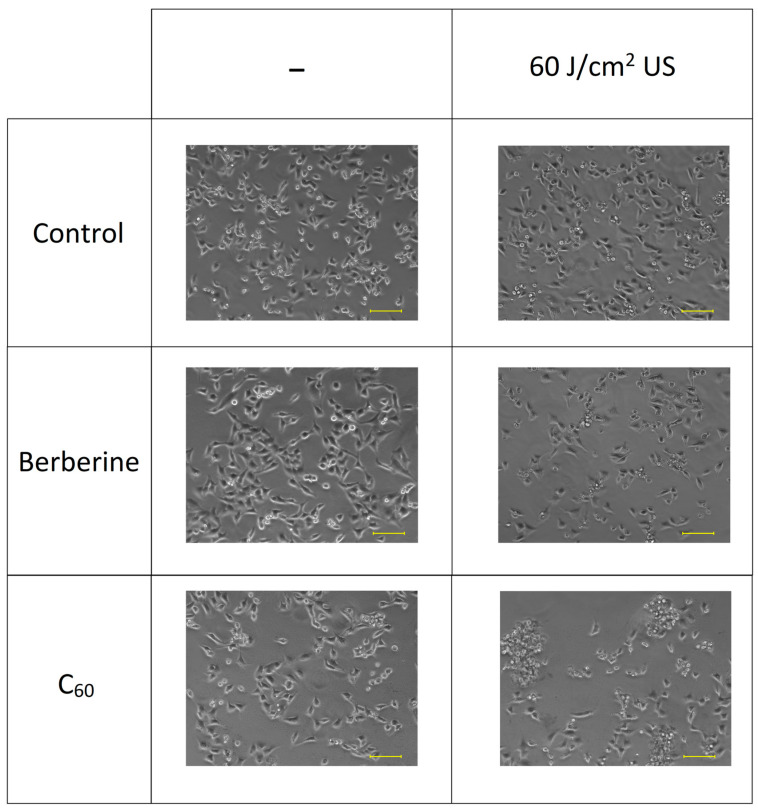
Phase contrast microscopy images of HeLa cells incubated in the presence of 20 µM C_60_ or 20 µM berberine and treated with 1 MHz ultrasound (US) in the “transducer in well” set-up; scale bar is 100 µm.

The “transducer in well” set-up demonstrated a more efficient decrease in cancer cell viability in combination with sonosensitizers at lower US power. Thus, A549, LLC, HeLa, and H4llE cell viability was decreased to 59 ± 3%, 56 ± 2%, 52 ± 2%, and 47 ± 2%, respectively, with the US dose of 60 J/cm^2^ in the presence of 20 µM C_60_, while sonicated berberine in the “transducer in well” set-up decreased cell viability to 65 ± 5%, 65 ± 2%, 57 ± 2%, and 60 ± 4% for A549, LLC, HeLa, and H4llE cells, respectively (Figure 7). In addition, morphological changes such as decreased cell density and apoptotic bodies were observed with phase contrast microscopy after treatment with sonosensitizers and 60 J/cm^2^ US, whereas 60 J/cm^2^ US without sonosensitizer did not affect cells (Figure 8).

Cells exposed to a combined treatment of 1 MHz US and 20 µM sonosensitizers demonstrated a considerable viability decrease. C_60_ showed higher sonocytotoxicity against all tested cell lines than berberine. In addition, HeLa and H4llE cells had higher sensitivity to treatment with C_60_ and berberine than other cell lines. Nevertheless, the “transducer in well” set-up demonstrated a higher cytotoxic effect towards A549, LLC, HeLa, and H4llE cells treated with C_60_ and 60 J/cm^2^ by 6%, 9%, 5%, and 13%, respectively, than the “sealed well” set-up. HeLa demonstrated higher sensitivity to the proposed treatment among human cell lines. Meanwhile, lung cancer is the leading cause of death among other types of tumor disease, and LLC has demonstrated significant sensitivity to sonodynamic treatment [2]. Therefore, LLC and HeLa cells were selected for comparison of intracellular effects as carcinoma models of different origins.

The data revealed C_60_’s and berberine’s ability to decrease cancer cell viability after sonoexcitation with 1 MHz US in both set-ups. Meanwhile, C_60_ demonstrated higher cell viability inhibition than berberine under US action. The obtained treatment cytotoxicity tendencies correlated with sonoluminescence intensity data at tested US exposure set-ups (Figure 6 and Figure 7). The US dose increased results in higher sonoluminescence intensity and cytotoxic effect towards treated cells, suggesting the sonoluminescence-dependent induction of sonosensitizer’s cytotoxicity. The spectrum of sonoluminescence consists of emission at 280, 310, and 340 nm [73,74]. C_60_ has three intense absorption bands, with maxima around 215, 265, and 350 nm [49,53]. Meanwhile, the absorption spectrum of berberine exhibits three bands, with maxima around 250, 350, and 420 nm [43,75]. The sonoluminescence spectrum matches the first long-waved maxima of C_60_ in a broader range than berberine, suggesting that it could induce the cytotoxic photosensitizing activity of C_60_ more efficiently. US without sonosensitizers showed no significant cytotoxic effect on cells at a dose ≤60 J/cm^2^. The highest ratio of cell viability between treated cells and corresponding control was observed at 60 J/cm^2^ in both set-ups. The “transducer in well” set-up demonstrated higher efficacy at 6–13% for A549, LLC, HeLa, and H4llE cells than the “sealed well” set-up. It was consistent with the US pressure and intensity distribution data, which showed higher sound pressure and temporal average intensity in the “transducer in well” than in the “sealed well” set-up.

The obtained results evidenced that 1 MHz US induced significant cytotoxic effects of proposed sonosensitizers against cancer cells. The inhibition of cell viability underlined the potential for the application of the proposed US set-ups to test sonosensitizing candidates for anticancer therapy. Similar experiments were performed by Tsuru et al., using a “transducer in well” set-up. Sixteen cell lines were treated with 7,12-bis(1-(2-(2-hydroxyethoxy)ethoxy)ethyl)-3,8,13,17-tetramethylporphyrin-2,18-dipropionatomanganese (DEG) and 1 MHz 1 W/cm^2^ US for 120 s. As a result, the cytotoxic effect varied from 52% to 87% [29]. Meanwhile, after 180 s of 1.93 MHz 6 W/cm^2^ US sonication in a “sealed well” set-up, the cell viability of human promyelocytic leukemia HL-60 treated with polyhydroxy C_60_ decreased to 56% [37]. Because of minor reflections and no undesired parameter variations, the “sealed well” set-up was one of the preferable US therapy set-ups for in vitro application [41]. 

Meanwhile, the main advantages of the “transducer in well” set-up include ease of positioning the transducer directly in the well and reduced wave reflections [42]. Lee et al. used human ovarian cancer SKOV-3 spheroids adhered to LP-9 normal mesothelial cell monolayers and treated them with pegylated graphene nanoribbons conjugated with chlorin e6. After 30 s of 1 MHz 0.8 W/cm^2^ US irradiation, complete cell death of SKOV-3 spheroids was detected, while LP-9 cells were not significantly affected after US irradiation [35]. Using a “transducer in well” set-up, Lee et al. showed many significant effects with less US output power than Yumita et al. showed with a “sealed well” set-up. However, comparing the research is complicated due to different models, cell lines, and sonosensitizers. Although (for experiments with a “transducer in well” set-up”) sterile conditions such as a laminar flow box are required, a “transducer in well” set-up can be easily standardized and more reproducible in comparison to a “sealed well” set-up [42].

The “transducer in well” set-up can demonstrate higher efficacy and reproducibility than the “sealed well” set-up. The US therapy unit provides a user-friendly cost-effective treatment strategy for translational research with animal models, as in the “transducer in well” set-up. Transducers of the US therapy unit could be aimed at the tumor location during the treatment procedure. Therefore, the “transducer in well” set-up was chosen to estimate apoptosis cell-death type induction.

### 3.5. Apoptosis Induction

To estimate further cellular effects, LLC and HeLa cells were incubated with 20 µM C_60_ or 20 µM berberine for 24 h, then irradiated with 1 MHz, 1 W/cm^2^ US for 30, 60, and 90 s in the “transducer in well” set-up.

**Figure 9:**
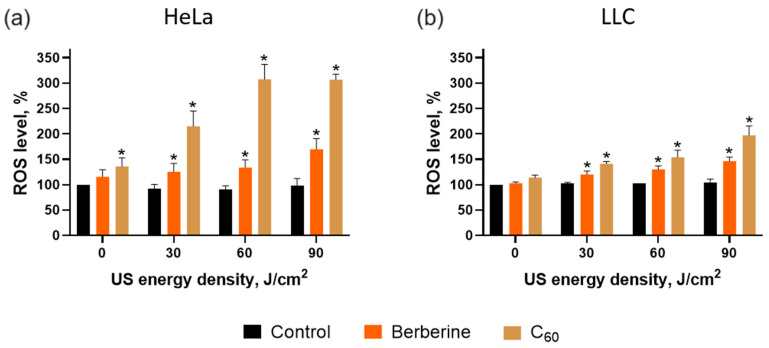
ROS level of Hela (**a**) and LLC (**b**) cells incubated in the presence of 20 µM C_60_ or 20 µM berberine and treated with 1 MHz ultrasound (US) in the “transducer in well” set-up; *—*p* ≤ 0.01 in comparison with the viability of cells, treated with the respective duration of US.

The efficient and continuous intracellular ROS production is critical for realizing the sonosensitizer’s anticancer effect [35,36,37,49]. Therefore, ROS generation was estimated with the ROS-sensitive fluorescence dye DHE. The ROS production was estimated 2 h after US exposure. The US irradiation insignificantly affected ROS levels in treated HeLa and LLC cells (Figure 9). After sonoexcitation of cells treated with 60 J/cm^2^ US and C_60_, ROS levels reached 307 ± 20% and 158 ± 22% in HeLa and LLC cells, correspondingly. Berberine demonstrated lower ROS generation, with 134 ± 14% and 130 ± 11% ROS level increases in Hela and LLC cells, respectively.

ROS level measurements revealed higher sonodynamic treatment efficacy toward HeLa cells, as the ROS level of LLC cells was lower by 173% and 28% for US with C_60_ and berberine, respectively. The obtained data can be linked to increased activity of the antioxidant system in lung cells related to the nuclear factor erythroid 2-related factor 2 (NRF2) [67,68,76].

ROS level increase leads to the loss of mitochondrial membrane integrity and cytochrome c release for further apoptosome formation. In comparison, apoptosome is an Apaf-1-containing complex that catalyzes the activation of caspases 3/7 [77]. Therefore, the ATP level and caspase 3/7 activity were investigated as markers of apoptotic cell death.

**Figure 10:**
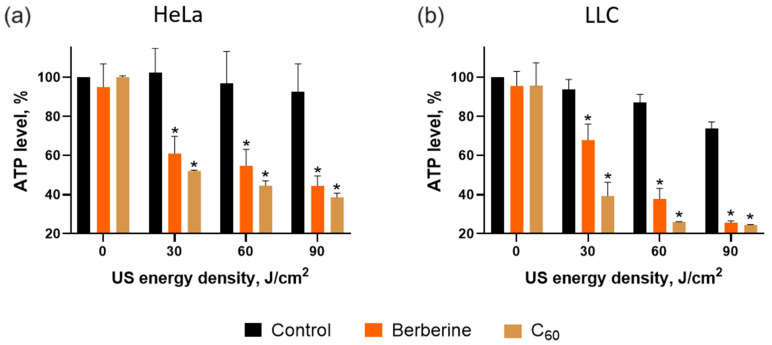
ATP level of Hela (**a**) and LLC (**b**) cells incubated in the presence of 20 µM C_60_ or 20 µM berberine and treated with 1 MHz ultrasound (US) in the “transducer in well” set-up; *—*p* ≤ 0.01 in comparison with the viability of cells, treated with the respective duration of US.

The ATP level was assessed as a principal marker of mitochondrial activity. The ATP level was measured 4 h after US treatment. Neither C_60_, berberine, nor US affected the ATP level in the cancer cells. In contrast, the sonication at 60 J/cm^2^ of HeLa and LLC cells treated with 20 µM C_60_ caused a decrease in the ATP level to 44 ± 2% and 26 ± 1%, respectively (Figure 10). Meanwhile, berberine induced the ATP level to drop to 55 ± 5% and 38 ± 5% in HeLa and LLC cells, respectively, after sonication at 60 J/cm^2^.

The decrease in ATP levels evidenced the mitochondrial toxicity of the sonodynamic treatment of cancer cells. As apoptosis is an ATP-dependent process, a lack of ATP could lead to unprogrammed necrotic cell death and an inflammatory process. Apoptosis is the most favorable process of programmed, ATP-dependent, and autonomous cellular dismantling that avoids eliciting inflammation [77,78]. Therefore, as a total ATP drop was not evidenced, apoptotic cell death could be executed.

**Figure 11:**
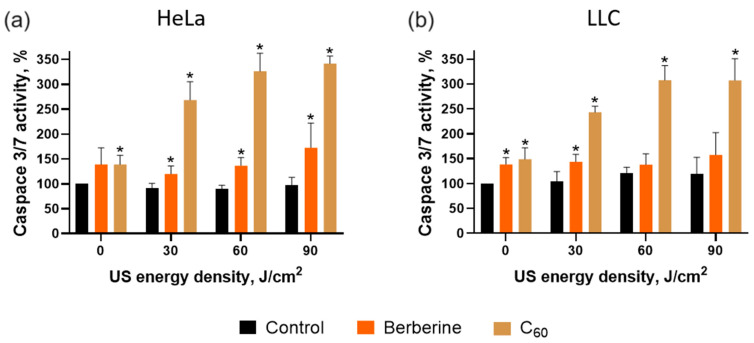
Caspase 3/7 activity of Hela (**a**) and LLC (**b**) cells incubated in the presence of 20 µM C_60_ or 20 µM berberine and treated with 1 MHz ultrasound (US) using “transducer in well” set-up; *—*p* ≤ 0.01 in comparison with the viability of cells, treated with the respective duration of US.

Activated caspase 3/7 executes apoptosis [77]. Therefore, the proapoptotic ability of the combinative treatment of C_60_ and berberine with 1 MHz, 1 W/cm^2^ US towards cancer cells was studied by evaluating the caspase 3/7 activity as a primary marker of apoptosis. Caspase 3/7 activity in treated cancer cells was estimated 4 h after sonication with 1 MHz, 1 W/cm^2^ US. It was shown that US did not affect caspase 3/7 activity. Meanwhile, the sonodynamic treatment with C_60_ and 60 J/cm^2^ US increased caspase 3/7 activity to 326 ± 23% and 307 ± 24% in Hela and LLC cells, respectively (Figure 11). Berberine demonstrated lower caspase 3/7 activity induction under US action, reaching 136 ± 12% and 138 ± 18% in Hela and LLC cells, respectively.

Since the employment of fullerenes, as well as SDT itself, for cancer treatment is still at the early stages of development, high attention should be paid to an identification of its possible toxicity and safety.

Our previous studies were focused on the evaluation of possible effects of C_60_ aqueous colloid solution on normal cells such as human primary keratinocytes hMSC cells, nondifferentiated rat phencocytoma PC-12 neuronallike cells, normal human mammary epithelial MCF10A cells, baby hamster kidney BHK-21 cells, and human embryonic kidney HEK293 cells. Thus, Tolkachov M. et al. showed that 6, 12, 18, and 24 μg/mL C_60_ did not affect the cell viability of hMSC cells for up to 24 h of incubation [79]. Prylutska S. et al. described the absence of the cytotoxic activity of C_60_ in a concentration range of 3.6–144 mg/mL against HEK293 cells at 24 h [80]. In addition, toxicity against MCF10A, PC-12, hMSC, and BHK-21 cells were described by Levi N. et al., Larner S. et al., Scrivens W. et al., and Liu S. et al., accordingly [81,82,83,84]. Summarizing the literature and our previous data, we conclude that the proposed pristine C_60_ aqueous colloid solution could be defined as nontoxic at the used concentrations towards normal cells.

Concerning the herbal alkaloid berberine, safety concerns should be evaluated as well. Published articles report that the safe dose of berberine in vivo for oral administration in mice is 20.8 g/kg of body weight, while intravenously it is 0.38 μg/mL [85]. Cytotoxic concentration of berberine towards normal cells varies from 47 to 293 μM [86], which is at least two times more than the used concentration in this research (20 μM), suggesting its applicability.

Several studies have reported SDT to be safe for normal cells and tissues. Lagneaux et al. showed that leukemia cells were damaged by 1.8 MHz 0.22 W/cm^2^ US, while normal hematopoietic cells remained unaffected [87]. Other published data reporting that US had no effect towards normal endothelial cell lines, while tumor cells were significantly affected [88]. Ohmura et al. demonstrated that the normal rat brain tissue was unaffected under long-term US with an intensity of 10 W/cm^2^ [89].

Our previous research evidenced the selective toxicity of C_60_-based SDT towards cancer cells. The treatment of normal human embryonic lung HEL 299 cells with 1 MHz US in the presence of C_60_ led to no significant decrease in the cells’ viability. Meanwhile, 60 s US treatment of HeLa cells in the presence of 20 µM C_60_ decreased cell viability to 59 ± 5% [36]. Also, US (1.86 MHz, 1.5 W/cm^2^, 300 s) treatment with porphyrinato palladium tetrachloride had no effect on the viability of human primary dermal fibroblast HDF 106-05 cells, while it led to a dramatic decrease in human colon cancer cell HT-29 viability [90]. Lee et al. demonstrated significant toxicity of SDT with pegylated graphene nanoribbons conjugated with chlorin e6 towards human ovarian cancer SKOV-3 spheroids. Meanwhile, an LP-9 normal mesothelial cell monolayer was not significantly affected after similar SDT treatment [35]. Therefore, we can assume that STD with sonosensitizers has selective toxicity against cancer cells, whereas the effect on normal cells is expected to be negligible.

The potential for using fullerene- or herbal alkaloid-based sonosensitizers in cancer SDT treatment protocols is substantial. Promising studies that have emerged in the last years have tested its validity and safety. The pharmacodynamics and pharmacokinetics of any new drug formulation should certainly be defined both in vitro and in vivo for an effective assessment of possible human health risks. Further research on the safety profile and treatment efficacy of the proposed treatment is ongoing.

## 4. Conclusions

The obtained data evidenced a toxicity of the tested sonodynamic treatment of LLC and HeLa cells with 1 MHz US and C_60_ or berberine via oxidative stress and apoptosis induction. Combining 1 W/cm^2^ US with C_60_ and berberine provided a promising platform for a synergetic approach to cancer treatment. Obtained data on ROS and ATP levels and caspase 3/7 activity underlined a suitable application of the “transducer in well” set-up for sonodynamic cancer therapy evaluation in vitro and pointed to the perspective of its application for further cancer SDT development.

## Figures and Tables

**Table 1:** SDT in vitro set-ups and treatment models.

US Exposure Set-Up	Sonosensitizers	In Vitro Model	US Treatment	Year of Publication	Reference
“well on transduce”	Polypyrrole-coated carbon nanotubes	Melanoma C540	1 MHz 1.0 W/cm^2^ non defined	2020	[33]
	Ferrite/carbon nanocomposite	Melanoma C540	1.0 MHz 1.0 W/cm^2^ 60 s	2019	[30]
	Fullerene covalently bonded onto the surface of black phosphorus nanosheets	Murine breast cancer 4T1	1.0 MHz 1.0 W/cm^2^ 300 s	2021	[39]
	TiO_2_ conjugated to polyethylene glycol	Glioblastoma U251	1.0 MHz 1.0 W/cm^2^ 50 s	2011	[31]
“well, on the water’s surface”	Polyhydroxy C_60_	Sarcoma 180	1.92 MHz 4.5 W/cm^2^ 60 s	2013	[38]
	C-doped TiO_2_	Murine breast cancer 4T1	1.0 MHz 1.8 W/cm^2^ 90 s	2020	[32]
	C_60_ aqueous solution	Human cervix adenocarcinoma HeLa	1.0 MHz 5.4 W/cm^2^ 60 s	2023	[36]
“sealed well”	Protohemin-conjugated multiwalled carbon nanotubes with carboxylic groups	Hepatocellular carcinoma HepG-2	1.0 MHz 0.5 W/cm^2^ 100 s	2016	[34]
	Polyhydroxy C_60_	Human promyelocytic leukemia HL-60	1.93 MHz 6 W/cm^2^ 180 s	2016	[37]
“transducer in well”	Pegylated graphene nanoribbons conjugated with chlorin e6	Human ovarian cancer SKOV-3	1.0 MHz 0.8 W/cm^2^ 30 s	2011	[35]
	DEG (7,12-bis(1-(2-(2-hydroxyethoxy)ethoxy)ethyl)-3,8,13,17-tetramethylporphyrin-2,18-dipropionatomanganese)	Human lung cancers LU65A, HLC-1, KNS-62; human gastric cancers MKN-1, MKN-28, MKN-45, MKN-74; human pancreatic cancers QGP-1 MIA PaCa-2; human prostate cancer PC-3 A human liver cancer KIM-1Human colon cancers HT-29, T-84, Caco-2; human breast cancers SK-BR-3, MCF-7	1.0 MHz 1.0 W/cm^2^ 120 s	2012	[29]

**Table 2:** Sound pressure and temporal average intensity.

Set-Up	Generator Output Power, W/cm^2^	Average Sound Pressure, kPa	Average Temporal Intensity, W/cm^2^
“sealed well”	1.1	179	1.1
	1.6	220	1.7
	2.2	258	2.3
“transducer in well”	1.0	450	7.0
	3.0	520	9.2

## Data Availability

The datasets used and analyzed during the current study are available from the corresponding author upon reasonable request.
